# A model of purchase intention of complementary and alternative medicines: the role of social media influencers’ endorsements

**DOI:** 10.1186/s12906-023-04285-1

**Published:** 2023-12-05

**Authors:** Gizem Gülpınar, Mehmet Barlas Uzun, Ayesha Iqbal, Claire Anderson, Wajid Syed, Mahmood Basil A. Al-Rawi

**Affiliations:** 1 Department of Pharmacy Management, Faculty of Pharmacy, Gazi University, 06330 Ankara, Turkey; 2 Department of Pharmacy Management, Faculty of Gülhane Pharmacy, Sağlık Bilimleri University, 06018 Ankara, Turkey; 3 Division of Pharmacy Practice and Policy, School of Pharmacy, University of Nottingham, Nottingham, NG7 2RD UK; 4 Office of Lifelong Learning and the Physician Learning Program, Faculty of Medicine and Dentistry, University of Alberta, T6G1C9, Edmonton, AB Canada; 5 Department of Clinical Pharmacy, College of Pharmacy, King Saud University, Riyadh, Saudi Arabia; 6 Department of Optometry, College of Applied Medical Sciences, King Saud University, Riyadh, Saudi Arabia

**Keywords:** Social media influencer, Complementary therapies, Source credibility, Health products, Theory of planned behavior, Complementary and alternative medicines

## Abstract

**Background:**

Social Media Influencers (SMIs) are a fashionable way of marketing products by creating electronic word-of-mouth (e-WOM) on social media. The marketing of complementary and alternative medicines (CAMs) by SMIs is becoming increasingly popular and gaining credibility within consumers on social media platforms. Nonetheless, advising about healthcare products on social media should be examined as it is different from endorsing other kinds of commercial products. The aim of this study is to develop a model that provides the underlying mechanisms of the stimuli of SMIs on social media towards consumers’ purchase intention of CAMs.

**Methods:**

This study used best fit framework synthesis methods to develop the model. A priori theory selection was conducted by identifying a BeHEMoTh strategy (Behavior of Interest, Health context, Exclusions and Models or Theories) to systematically approach identifying relevant models and theories relative to the research aim. Further evidence derived from primary research studies that describe the behavior identified is coded against selected a priori theory to develop the model.

**Results:**

This study presents a novel model for understanding the purchase behavior of CAMs using SMIs as a marketing strategy. The model included two well-known theories (theory of planned behaviour theory and source credibility theory) as well as extensive existing research from a multidisciplinary perspective. The model is exclusively designed to help identify elements affecting perceived source credibility and factors that have an influence over consumers’ preferences to purchase CAMs by taking into consideration SMIs’ endorsements.

**Conclusions:**

This study provides unique insights introducing new research areas to health literature and offers, new roles for healthcare professionals in this digital era by gaining new skills and competencies required to provide more credible and accurate information about CAMs. The study also highlights the new marketing era of online health-related product endorsements and recommends that policymakers and researchers carefully evaluate the impact of SMI’s on the use of CAMs, as well as to regulate the content of these promotional materials.

**Supplementary Information:**

The online version contains supplementary material available at 10.1186/s12906-023-04285-1.

## Background

Consumers are increasingly using social media to gather information on which to base their decisions to purchase products. There are people on social media platforms called social media influencers/opinion leaders (SMI’s) who are known to hold a certain level of influence over other people [1, 2]. Brand marketers have accelerated to approach (SMIs) to e promote and endorse their products [3]. This has given rise to; a new trend in marketing commonly called “influencer marketing” by generating e-WOM (electronic word-of-mouth) [4].

e-WOM is a behavior through which the message is diffused among the consumers, whereas viral marketing is a technique creating “viral infection” or “buzz” used by companies [5, 6]. Consumers who spread awareness about product or service offerings among their social networks by using e-WOM behavior, highlight pros and cons to assist other consumers with buying decisions [7, 8]. Conventionally, traditional marketing focused on using famous celebrities, TV and film stars, for marketing their brands, because people like and trust the advice, of people whom they like [9]. With the social media era, the brands and marketers realized that SMI’s on commonly used social media platforms [10] such as Meta, Instagram, twitter, tik-Tok also have a lot of people following them because they are considered influential members of the community. SMI’s usually create an “influence” over consumers by creating e-WOM messages using textual, verbal as well as video content [11].

Despite the fact that existing literature has explored the effects of SMIs on various commercial products and services [1–3, 12–15], there remains a noticeable gap in the literature concerning their impact on health-related products, particularly CAMs [16]. CAMs cover a diverse array of natural and herbal products, each offering unique modalities and practices. Over the years, interest in and use of CAMs have gained popularity among individuals seeking complementary or alternative treatments to conventional medicine [17]. With the emergence of social media, there is now a medium for attaining health information as well as purchasing products and services relative to CAMs. Although existing literature provides insights into the motivations behind CAM usage [18, 19], the role of SMIs in shaping consumer decision-making behavior regarding CAMs has remained largely unexplored. While some studies touch upon the marketing of health products through social media channels [8, 12, 15, 20, 21], a comprehensive understanding of the influencing factors that drive consumers to purchase CAMs through SMIs’ endorsements is yet to be established.

Advising about health-related products, including OTC medicines, medical devices, and complementary and alternative medicines (CAMs), is far different than marketing or endorsing travel destinations or convincing people to buy a box of toys. Nevertheless, for example, the pharmaceutical and nutraceuticals industry have recognized the potential power of Instagram as a social media marketing platform by assigning SMIs to market their healthcare products, creating e-WOM, which has had the tendency to increase their product sales and generate business [13]. To that said, studies exploring influencers’ narrative strategies for various brands and industries are expanding for the advancement of social media marketing [22–24]. However, the development of knowledge regarding the strategies for influencers to attract consumers to be used by the pharmaceutical industry is still in need.

Taking all this into account, there is both a managerial and an academic need to better understand the role played by SMIs in the pharmaceutical industry, especially for CAMs. To bridge the existing research gap between the motivational factors (cognitive process, emotions, and individual factors) behind CAM usage and the influence of SMI stimuli on consumers’ purchase behavior concerning CAMs, the present study seeks to develop a comprehensive model. This model will shed light on the intricate interplay between these two critical aspects, providing valuable insights into consumers’ decision-making behavior regarding CAMs on social media platforms. Our research aims to model the underlying mechanisms that shape consumer choices by providing insights into SMIs’ endorsements and motivational factors of CAM usage. Through this holistic approach, we hope to contribute significant knowledge that empowers both scholars and marketers to better understand the combined impact of motivational factors and SMI stimuli on consumers’ CAM purchase behavior in the digital age.

## Methods

**Fig. 1:**
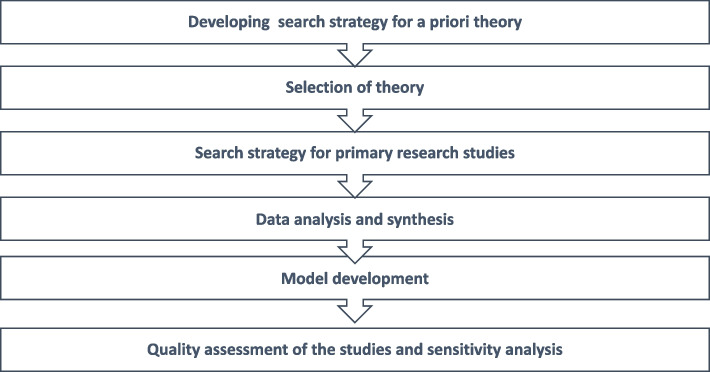
Overview of methodology [26]

This study used “best fit” framework synthesis approach [25, 26] for evidence synthesis to develop a Complementary and Alternative Medicine Purchase Intention Model (CAMPIM). This approach enables the authors to systematically identify relevant frameworks, models, or theories for selecting a priori theories with the “best fit” to the topic. Further evidence derived from primary research studies that describe the influence of SMI on consumers’ purchase behavior of CAM and the factors affecting people purchasing CAM was coded against selected a-priori theories. An overview of methods used in this study is shown in Fig. 1.

### Developing a search strategy for a priori theory

The framework synthesis included identifying a BeHEMoTh strategy [26] (Behavior of Interest, Health context, Exclusions and Models or Theories) to systematically approach identifying relevant models and theories, relative to the research aims as shown in Table 1. Databases included were MEDLINE (OVID), Embase (OVID), PsycINFO (Ovid), CINAHL (EBSCO), Science Direct, Emerald, JSTOR, Business Source Ultimate, ProQuest. All study designs, including theories and models to describe the behavior presented in Table 1, were included in this study, however, no grey literature was included. The bibliography of selected papers was also reviewed systematically, as well as Google Scholar searches to find relevant information in similar studies.

**Table 1:** BeHEMoTh strategy for framework analysis

Components of the BeHEMoTh strategy	Taxonomy for this research study
Be: Behavior	• The influence of SMI marketing on consumers’ purchase behavior • The influence of SMI marketing on consumers’ purchase behavior of CAM • Understanding why/how people purchase CAM
H: Health Context	Chronic conditions management
E: Exclusions	CAM used for; mental health conditions, studies informing parents’ behavior to purchase CAM for children or studies reporting findings below 18 years of age.
MoTh - Models or Theories	Model or theory or theories or framework or concept or conceptual relating to explaining the influence of SMI marketing on the purchase behavior of CAM

### Selection of theory

Two independent authors (Author 1 and 2) searched and identified relevant theories relating to the research aims. After their initial assessments, both authors discussed the merits and demerits of each selected theory via regular meetings via Microsoft Teams (USA) after being retrieved from the databases described in the previous section. Consensus was reached on theories to be used as a-priori theory representing the core ideas of the research aim.

### Search strategy for primary research studies

To identify relevant primary research studies, clear research questions were developed as shown below:

- What kind of information people look up on social media about CAMs?
- What motivates people to use CAMs and purchase CAMs online?
- What do people look for/in SMIs? Why do people trust SMIs?
- On what criteria do people select influencers and then adhere to their information?
- What type of strategies do SMIs use while marketing CAM products or other products?

**Fig. 2:**
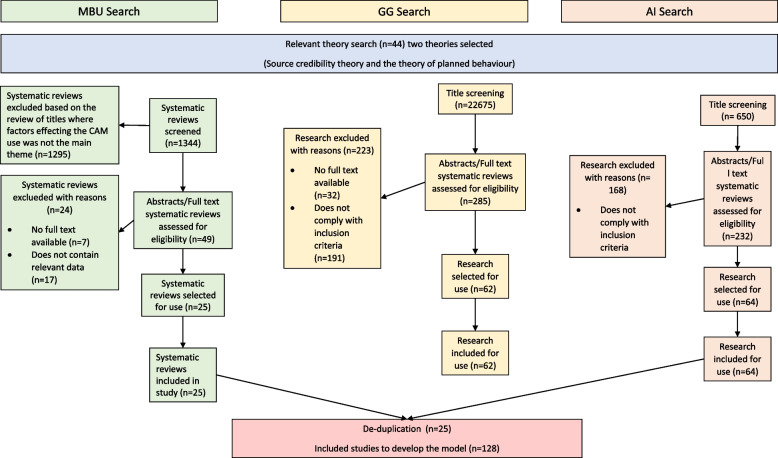
Search results for primary research studies

These questions led to the development of individual search strategies using relevant taxonomy and keywords appropriate to each question (Appendix 1) and involved conducting searches using selected databases. MEDLINE (OVID), Embase (OVID), PsycINFO (Ovid), CINAHL (EBSCO), Science Direct, Emerald, JSTOR, Business Source Ultimate, ProQuest were searched by three authors. Author 1, 2, and 3 undertook an independent search using an assigned research question, respectively (Fig. 2). All study designs were included in this study; however, no grey literature was included. The bibliography of selected papers was also reviewed systematically, andGoogle Scholar searches were done to find relevant information in similar studies. Backward citation tracking was also used to trace more studies. The search was limited to articles available in the English language or English translation. The studies included the use, or intention to purchase CAMs and SMI influences only in adult population. The inclusion and exclusion criteria of inclusion of this study are also provided in Table 1. The final inclusion of primary research studies was discussed and approved by all authors. Relevant studies were exported to Endnote X9 (Clarivate Analytics, PA, USA), and duplicates were removed.

### Data analysis and synthesis

The approach described by Carroll et al. [26] was adopted for the data analysis and synthesis. After the studies were selected, a data extraction form was developed that included the main constructs of the a priori theories [27]. In this way, the data were coded using the data extraction sheet following a deductive approach. The data in the studies were extracted from the methods, results, and discussion sections of the selected studies. Two researchers (Authors 1 and 2) independently coded two studies to agree on the final form. Relevant data from primary research were coded into themes (sub-constructs of the model) and sub-themes [28]. This initial coding was then supplemented by secondary thematic analysis to capture the remaining evidence that did not fit the data extraction form. The themes were then discussed by the research team, and when necessary, new themes were developed that fit the codes uncovered by the a priori theories. Finally, the author team agreed upon the final list of themes and sub-themes.

### Model development

Relationships between the themes of the model were recreated or generated based on the evidence from the primary research studies included in this study by focusing on social explanations developed from comparative understanding. This also involved the process of translation [29], which helped bring together themes from different studies to become representative of each other. This step helped provide a robust testing process and implying transferability or inferential generalization. The final model development involved multiple synthesis and refinement cycles as well as developing a consensus on the taxonomy of constructs used within the model by all authors.

**Fig. 3:**
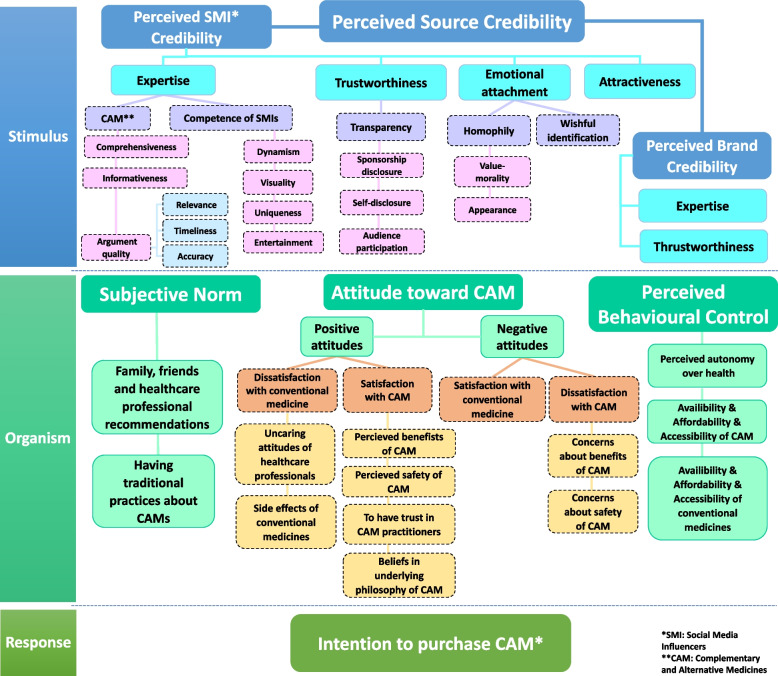
The complementary and alternative medicine purchase intention model

Stimulus-Organism-Response (SOR) model was used to help visualize and provide the connectivity in two selected a priori theories in Fig. 3. The SOR model is a psychological framework used to understand the process of human response to stimuli in a given environment [30]. It recognizes that human behavior is not solely determined by external stimuli but also by internal factors such as cognitive processes, emotions, and individual differences. Thus, the part of the model derived from the Source Credibility theory modeling the factors of how SMIs influence consumers to purchase CAMs represents the stimulus, whereas the part derived from the TPB that explains the cognitive, emotional, and individual factors of why consumers use CAMs refers to the organism part of the SOR. Finally, response refers to the intention to purchase CAM in the model (see Fig. 3).

### Quality assessment of the studies and sensitivity analysis

Author 1, 2, and 3 conducted independent quality assessments of the included studies. The focus was on the reporting of basic methods and not potentially subjective judgments regarding studies’ validity or reliability. Although the presence of uncertainty in the quality of a study’s execution is recognized, providing a clear description of the authors’ methodology, including approach, sampling, data collection, and analysis methods, can enhance the strength and reliability of the study’s outcomes [31]. This does not preclude the possibility that an “inadequately-reported” study has actually been well conducted, but it does form a reasonable basis for making a quality assessment. Studies were deemed adequately reported if they offered comprehensive information on two or more criteria. A sensitivity analysis would be performed in the event of the inclusion of “inadequately-reported” studies [31, 32]. A sensitivity analysis [32] aims to investigate whether the results were significantly impacted by inadequately-reported studies or other specific characteristics. In other words, whether any of the themes generated in the data analysis were lost because of the exclusion of these studies would be evaluated.

## Results

### Study selection for a-priori theories, primary research studies and quality assessment

The BeHEMoTh search approach focusing on theories regarding consumers’ purchase behavior of CAMs and the impact of SMI stimuli on consumers’ purchase behavior resulted in 44 studies (Fig. 2). Twelve eligible theories and models were identified, but the theories with rich information and concepts reflecting major aspects of the research aim were focused on. Two theories were chosen as a priori theories for this study. Theory of Planned Behavior (TPB) [33–36] and Source Credibility theory [37, 38]. The TPB is a cognitive theory developed by Azjen (1985) which proposes and provides explanations to an individual’s decision to engage in a specific behavior. TPB operates within three main constructs: behavioral beliefs, normative beliefs, control beliefs [36]. This theory provided key components for the initial framework to understand the intention drivers for behavior to purchase CAMs. Source Credibility theory developed by Hovland et.al (1940) states that people are more likely to be persuaded when the source presents itself as credible [37, 38]. The credibility of all communication, regardless of the format it is being delivered in, has been found to be heavily influenced by the perceived credibility of the source of that communication. This theory was selected because it helped inform source credibility of SMIs using social media sites. Details of other relevant theories and models identified can be found in Appendix 2.

For primary research studies, 128 studies were used to develop the model by combining all the searches by three authors (Author 1, 2, and 3) as shown in Fig. 2. No study failed to describe clearly at least two of the following: the question and study design, and the methods of sampling, data collection or analysis. Hence, a sensitivity analysis was not performed.

The following section now presents an overview of the main themes of the model, the dotted boxes depicted in Fig. 3 are the sub-themes of stimulus part (derived from source credibility theory) and organism part (derived from TPB) of the model and are explained in Tables 2 and 3 respectively.

**Table 2:** Conceptual definitions of sub-themes developed in the stimulus part of the model

Themes	Sub-Themes (Dotted boxes in the model)	Concept
Expertise	Argument quality —Relevance —Timeliness —Accuracy	Argument quality’ refers to the persuasive strength of the information provided within a message provided by SMIs [39] It is related to how much consumers perceive the argument as convincing in defending its position [39, 40]. Relevance refers to the extent to which endorsements are applicable and useful for decision making [41]. Timeliness represents whether an endorsement is present, timely, and up-to-date [41, 42] Accuracy refers to endorsement/argument reliability. It also characterizes whether a receiver (customer) perceives the information within a message as correct [43]
	Comprehensiveness	Comprehensiveness refers to how delivered messages perceived by consumer with regards to its completeness [42].
	Informativeness	Informativeness refers to SMI’s ability to provide information about alternative products to boost consumers’ purchase satisfaction [44].
	Competence of SMIs —Dynamism —Uniqueness —Entertainment —Visuality —Popularity	Dynamism implies whether SMIs are responsive and active in creating content within their accounts [45]. Uniqueness refers to a situation in which a person feels distinguished from other people around them [46] and involves behaving in a manner that others will perceive this person as specific and different [47]. In consequence, consumers might admire the personal image created by an SMI due to being perceived as unique [48]. Entertainment and visuality refer to the extent to which SMIs stamp their posts with personal aesthetic touches (visuality) and personality twists, which generally generate an enjoyable experience (entertainment value) for their followers [2]. Popularity refers to the high number of followers [49]. People assess one’s popularity by considering the number of online contacts, followers, or friends [50]. When an SMI has a high number of followers, they are noticed as a credible source of information [50].
Trustworthiness	Transparency —Sponsorship disclosure —Self-disclosure —Audience participation	Transparency mainly refers to SMIs’ clarification about the motivation behind a promotional message by revealing bonds between contributors or emphasizing honest opinions [1, 51]. Sponsorship disclosure is a way to provide transparent communication about whether a post is marketed by a third party [52]. SMI trustworthiness and persuasion are influenced by the type of sponsorship disclosure due to transparency [51]. Self-disclosure refers to the process by which persons let themselves be known to others [53]. Self-disclosure includes “any information exchange that refers to the self, including personal states, dispositions, events in the past, and plans for the future” [53]. For example, on Instagram, users can make information public, meaning their profile and posts can be viewed by anyone interested in following them, or private, which include approved users or followers. Audience participation involves the interactions between an audience (consumer) and SMIs on posts, including liking or leaving comments. When consumer has an interest in being involved in posts, it offers possibilities for widespread message dissemination to peers within their networks [54].
Emotional attachment	Homophily —Attitude —Background —Value-morality —Appearance	Homophily concerns the degree of similarity among SMIs and consumers based on their beliefs, values, social status, and interests [55, 56]. When consumers believe that they share particular interests, values, or characteristics with an SMI, they are more likely to adopt their beliefs, attitudes, and behaviors [57]. The attitude dimension of homophily is the degree of similarity in attitudes (thinking, behavior). The background dimension includes a perceived degree of similarity in one’s social background (social, economic status, and social class). The degree of similarity between morals and values (personal morals and values, cultural values) forms the value dimension, while the appearance dimension reflects the degree of similarity in terms of visual attributes [58, 59].
	Wishful identification	Wishful identification refers to a desire to be like the other person [57]. In the case of social media, consumers mainly adopt SMIs’ behavior due to aspiration of being like the person they follow [60].
Attractiveness		Attractiveness focuses on an SMI’s physical attributes or characteristics [9]. An attractive SMI has the power to affect consumers with positive outcomes and subsequently with a purchasing intention.

**Table 3:** Conceptual definitions of sub-themes developed in the organism part of the model

Themes	Sub-Themes (Dotted boxes in the model)	Concept
Positive attitudes	Dissatisfaction with conventional medicines —*Uncaring attitudes of healthcare professionals* —*Side effects of conventional medicines*	The uncaring attitude of healthcare professionals and the side effects of conventional medicines may make individuals feel dissatisfied with conventional medicines. This dissatisfaction might lead consumers to adopt positive attitudes toward CAM. The causing factors of this dissatisfaction are described below as sub-themes depicted in the model: —*Uncaring attitudes of healthcare professionals:* Certain influences,such asthe lack of empathy of healthcare providers [18, 61–63], might lead people to use/find information on CAM of other people using CAM for similar problems in the hope of relating to a sense of attachment and empathy of experts in healthcare. These people were conventionally found in the wider community and social networks, but with the emergence of social media, the social media platforms now have become the new “social networks”, which hold the same power of influencing and exercising empathy [64]. The empathy needed by consumers could be found being demonstrated by SMIs. This might cause a shift in positive attitudes and being more receptive to SMIs, which in turn might influence their behaviors to purchasing CAMs. —*Side effects of conventional medicines:* Perceived side-effects of conventional medicines are the other factors found in studies [18, 61–63, 65] related to dissatisfaction with conventional medicines, and this was found to be an influencing factor leading people to purchase CAMs.
	Satisfaction with CAMs *—Perceived benefits of CAM* *—Perceived safety of CAM* *—To have trust in CAM practitioners* *—Beliefs in the underlying philosophy of CAM*	The same was found faithful in the opposite cases, where mutual satisfaction with CAMs, was found to influence a positive impact towards purchasing these products. Perceived benefits of CAMs, [18, 62, 65–69] perceived safety of CAMs, [18, 62, 63, 65, 67, 68, 70–72] trust in CAM practitioners, [63, 71, 73] positive beliefs in the underlying philosophy of CAM [63, 71, 73] were the factors which led to satisfaction with CAMs.
Negative attitudes	Dissatisfaction with CAMs *—Concerns about the benefits of CAMs* *—Concerns about the safety of CAMs*	Concerns about the benefits of CAMs [18, 74] and concerns about the safety of CAMs [18] have found the reasons for dissatisfaction with CAMs that lead consumers to adopt negative attitudes towards CAM.
	Satisfaction with conventional medicines	Mutual satisfaction with conventional medicines [18, 75] was found to have a negative impact on purchasing these products.

### Development of the complementary and alternative medicine purchase intention model

Figure 3 shows the CAMPIM model. Each concept (boxes in the Fig. 3) is now described along with its identified determinants and relationships. The “stimulus” part of the model depicted in Fig. 3 illustrates the themes and sub-themes showing the influence of SMIs on consumers’ purchase behavior of CAMs by using a-priori theory of Source Credibility, and the “organism” part of the model refers to the themes and sub-themes mapped onto TPB showing the factors affecting people to use CAMs directing them to adopt an intention to purchase CAMs.

### The stimulus part of the model derived from a-priori theory of source credibility

#### Perceived source credibility

Source credibility is a concept that means the more credible the source appears to seem, the more chances are the message or phenomenon will be accepted [38]. The credibility of the message source shows how much the recipient believes in the sender [76]. In the context of social media, source credibility is the measure to which content producers or SMI’s are perceived as trustable, having knowledge, and are considered ‘credible’ [41]. Source credibility was a significant factor found in studies that were seeking information about CAMs and the use of CAMs, was linked with the credibility of source (SMIs), advertising, selling, or giving advice to use CAMs [77]. Credible and perceived ‘accurate’ information provided by SMIs about CAMs are linked with how they approach these products and adjust and adhere to them.

Perceived SMI credibility and brand credibility are the two dimensions of source credibility generated as main themes in the model (Fig. 3) [2, 78] SMI’s own credibility refers to the extent to which to convey an audio or video endorsement about a particular product [2]. The reputation and credibility of the manufacturer or brand developing the product were also found linked with source credibility [78]. Source credibility has been conceptualized as having two main dimensions—trustworthiness and expertise [37]. These main dimensions are used for both SMI and brand credibility in the model. In addition, two additional themes developed from primary research studies were inserted in the model for SMI credibility (Fig. 3). The following section presents an overview of perceived SMI and brand credibility and their developed sub-themes.

#### Perceived brand credibility

Brand credibility derived from brand signaling theory proposes that markets are full of products and random information about these products. The information may be symmetric, asymmetric, focused, unfocused, company-created, marketeer-created, or the public sharing their experiences and perceptions of products [79, 80]. Amongst all this chaos in information, brands serve as signals of products based on their strong credibility of being authentic, and reliable, which in turns makes their products appear more credible [81]. Brands use different marketing and promotional techniques to create signals [80]. However, the credibility of brands comes when they build on their past successes and proven, promised products, and this is commonly known as ‘reputation’ in the economics literature [80].

Brand credibility thus broadly requires two core components: trustworthiness and expertise [80]. Brand credibility is defined as how effectively the product information is conveyed by the brand signal, which requires that consumers perceive that the brand has the ability (i.e., expertise) and willingness (i.e., trustworthiness) to deliver what has been promised continuously [82]. Credibility is linked to the confidence of consumers in that product or brand that they have the ability (i.e., expertise) and willingness (i.e., trustworthiness) to continuously deliver what has been promised [81]. Customers looking for CAM information or to be able to purchase CAM, and look for credibility of both the brand, company, manufacturer, distributor who are providing these products [83]. For example, consumers may tend to believe that a well-known brand will provide the promised level of effectiveness and safety of a CAM, whereas an equivalent claim by a less-known brand may be less credible.

#### Perceived SMI credibility

Perceived SMIs’ credibility is one of the factors determining the influencer endorsement effectiveness [84]. In the context of CAMs, this means that the more people who perceive an SMI is credible, the higher the chances that they will agree to the endorsement content and may continue to make the purchase [37, 38]. Studies reported that SMIs’ credibility consists of several dimensions –expertise, trustworthiness, and attractiveness [38, 84]. When consumers perceive the SMI to be an expert, trustworthy, and willing to provide accurate information, they might forgo the thinking process and, without thinking, accept their message as reliable and credible [8]. In terms of CAMs, consumers prefer sources that are more credible when they try to access the details of the product before making the purchase [85]. Moreover, emotional attachment is the other dimension added to the model [86]. Credible or even new SMIs (without established credibility but have some similarities with the followers) might develop and deliver content related to diseases and conditions, which might give people confidence and enable them to purchase of CAMs.

The below part presents the explanations of the main constructs of perceived SMI credibility (expertise, trustworthiness, attractiveness, and emotional attachment) in the model. In addition, Table 2 shows all the developed sub-themes shown in dotted boxes in Fig. 3 of perceived SMI credibility in the model and briefly provides the conceptual definitions of these sub-themes.

#### Expertise

Expertise refers to the knowledge, skills, or experience of a SMI. However, expertise in marketing literature examining influencers’ effect on decision-making process does not mean professional expertise, but rather, the expertise of the SMI to target their followers and people on social media to perceive them as experts [37, 38]. SMIs building their content on their actual expertise, or, for example, projecting themselves as experts on a specific genre, were found to be linked with more follower satisfaction [87]. A message coming from a perceived expert has a higher chance of producing a behavior change in consumers and leads to the purchase of products [9, 38]. Thus, the expertise of an SMI that is shown as CAM expertise in the model was anticipated to impact the purchasing of products. Social media studies report that the higher the informativeness [44], argument quality [39–42], and comprehensiveness [42] of the endorsement content provided by SMIs, the more people perceive them to be experts and believe in their endorsements as being credible. Hence, three sub-themes are developed for CAM expertise, defined in Table 2.

In addition to providing high-quality information about CAMs indicating CAM expertise, SMIs have some characteristics indicating their competencies that impact the consumers’ perceptions [47] that lead them to be considered experts. Table 2 presents conceptual definitions of every developed sub-theme of various competencies of SMIs in the model.

#### Trustworthiness

The motivation for including trustworthiness, the second theme of SMI credibility, in CAMPIM comes from the efforts of CAM consumers to acquire trustworthy advice [70]. As previously mentioned, studies reported that the use of CAM and adherence to CAM were linked to the lack of trust in prescribers due to their lack of empathy towards them [18, 61–63]. Social media studies reported that trustworthiness was a considerable criterion for people to place value on the influencer or endorser [4, 88, 89]. Having trust and belief that an SMI might project honesty, empathy, and demonstrate integrity in their information might inspire trustworthiness, which is directly linked to acceptance of their content, to be true [2]. Studies also reported that marketers and brands also invest in SMIs who are valued as trustworthy members of the audience [2, 90]. The overview of the sub-themes of trustworthiness in the model can be seen in Table 2.

#### Emotional attachment

Emotional attachment was found to be a strong factor in influencing people’s behavior [91, 92]. Emotional attachment, the third theme of SMI credibility in the model, means a bond between people and the SMIs. In social media, emotional attachment toward SMIs positively affects people to purchase a product, the SMI was endorsing [92]. Emotional attachment involves two aspects in the model: homophily [58] and wishful identification (for the definitions of the sub-themes, see Table 2) [93]. Emotional attachment with SMIs is usually inspired by content creators by building their ‘similarities’ to their target audience of the intended products usually by using similar beliefs, social status, interests, benefits, value addition and convenience.

Emotional attachment was also a construct identified in research regarding CAM, where people suffering from the same diseases and conditions feel emotionally attached and supported by using similar CAM products and forming beliefs (homophily in the model) [64]. The sense of emotional attachment was found to be a substantial factor in convincing others to influence CAM purchases [94]. Consumers might purchase CAMs because they desire to be like the other person they admire (wishful identification in the model), a SMI, where they already have developed emotional attachments. Thus, emotional attachment and perceived subjective norms can have a relationship in terms of whether consumers follow the suggestions of those similar to them within their social networks.

#### Attractiveness

Attractiveness in social media studies shows that an SMI, might be considered attractive if they are perceived by the followers as classy, sensual, and beautiful [1] and was found as another factor impacting consumer buying behavior [95]. In terms of the product content, the attractiveness of an SMI means that the information and endorsements can be trusted and agreed upon as credible if the SMIs themselves look attractive while consuming/applying/using them [89]. However, attractiveness in CAM studies was found to vary in context, where people who got better by using CAMs and returned to either everyday lives or manageable lives or even reporting improved quality of life were perceived as having a normal lifestyle, which seemed ‘attractive’ to people and people were attracted to. Although medical-related CAMs are not the beauty or cosmetic products that require physical attractiveness of an SMI to be purchased on social media, consumers could adopt SMIs’ advice when some unique traits, such as charisma, have been identified. This could lead to a purchase intention. Therefore, attractiveness is the fourth theme of SMI credibility developed.

### The organism part of the model derived from a-priori theory of TPB

This part explains the developed themes from primary research studies mapped onto the constructs of TPB: attitudes, subjective norms, and perceived behavioral control [34, 36]. Table 3 explains all the sub-themes depicted in dotted boxes in Fig. 3 corresponding to the organism part of the model.

#### Perceived subjective norm

What other people think and do is one concept that shapes our behavior [33]. The perceived subjective norm can be defined as “*beliefs about the normative expectations of others*” [35]. Normative beliefs thus include both; when an individual performs the behavior by observing (intrinsic drive) their social networks that leads them to perform the behavior in a particular ‘conventional’ way similar to others or the individual’s social networks directly and indirectly (extrinsic drive) prompting them to perform the particular behavior [19, 96].

Subjective norm in the model includes two themes that were found to impact intention. This part represents an overview of these themes and relationships with the model.

##### Having traditional practices

One of the roles that society assigns individuals is seeking and accessing healthcare services and/or products responsibly when they get ill [97]. Ideally, conventional medicine exists to help individuals to fulfill this task with scientifically proven treatments [98]. When this task cannot be met by conventional medicine due to several factors, such the existence of an incurable disease, lack of compliance with treatment, lack of communication, accessibility-availability-affordability problems [99], individuals begin to find the necessary treatment from other sources rather than conventional medicine such as CAMs. In addition, having been pre-exposed to cultural and traditional practices about using CAMs [18, 61, 66–68, 71, 100–104] are seen as influencing factors that drive the intention to purchase CAM.

##### Family, friends, and healthcare professionals’ recommendations

Studies reported that family and friends [18, 61, 65, 67–71, 73, 100, 102–104], physician recommendations [18, 61, 65, 70, 102, 103], are also external factors affecting individuals to purchase CAMs, which are the developed themes included in the model. In social media, posts about health-related experiences and searching for health information on the internet are increasing day by day [105]. It is inevitable for individuals to have subjective norms in social media, where there is a high level of interaction and messages open to others’ access [106].

#### Attitude towards CAM purchase

Attitude towards behavior refers to the degree to which a person has made an evaluation (can be favorable or unfavorable) of a particular behavior. According to TPB, behavioral beliefs and attitudes toward the ‘behavior’ are significantly linked. Each individual’s belief is related from the outset to achieving the certain behavioral outcome(s). These were found to influence an individual’s interest in believing and developing a positive attitude towards the behavior [33].

Regarding social media studies, people purchase products because other people are reporting to have purchased them [107]. Nowadays, specially Instagram is mostly being used for marketing purposes that allows people to leave their comments based on their evaluations and outcomes after using the products [15]. Hence, consumers could develop positive or negative attitudes and behaviours that help them decide whether to purchase the same products or look for different ones [44]. By investigating the literature, different perspectives were grouped under developing positive or negative attitudes for using CAM in the model, which could drive the behavior to purchase CAMs.

##### Positive attitudes toward CAM

The first sub-theme of positive attitudes toward CAM was the satisfaction and dissatisfaction of people with conventional medicines [18]. When people are satisfied with CAM usage and dissatisfied with conventional medicines for several reasons generated as sub-themes, they may be more likely to adopt positive attitudes toward CAM. This may drive their attitude and beliefs towards the purchase of CAMs. The detailed explanation of sub-themes of positive attitudes toward CAM can be seen in Table 3.

##### Negative attitudes toward CAM

The developed sub-themes of negative attitudes toward CAM in the model are the opposite cases of the positive attitudes (see Fig. 3). Satisfaction with conventional medicines and dissatisfaction with CAMs are seen as the reasons for adopting negative attitudes toward the intention of purchasing CAMs. The detailed explanation about sub-themes of positive attitudes toward CAM can be seen in Table 3.

#### Perceived behavioural control

Three main themes developed in the model refer to an individual’s perceived ease or difficulty of using or purchasing CAMs (see Fig. 3) [33, 35].

##### Perceived autonomy over health

Many CAM users seek these products because these products offer autonomy to them for decision-making and taking control of their health [63, 65, 70–72, 102, 108]. Purchasing CAMs by reflecting on SMIs’ endorsements could cause customers to think they have autonomy in selecting health products by themselves without any healthcare professionals’ advice.

##### Availability, affordability, and accessibility of CAM

Availability-accessibility-affordability of CAM [18, 65, 71, 74, 100] were considered as easing factors of using or purchasing CAMs in the literature. The ease of accessing health information [105], through SMIs could impact people’s intention to purchase these products by affecting consumers’ perceptions of availability and accessibility of CAMs. SMIs play a significant role in informing and contextualizing social media content for their followers. They do this by explaining the ease of use of these products and the overall simplicity of accessibility of CAMs by clicking and buying these products. Moreover, SMIs could show the relative benefit of CAMs over money to persuade consumers to believe these products are affordable [18].

##### Availability, affordability, and accessibility of conventional medicines

In most cases, availability-accessibility-affordability of conventional medicines [65, 70, 71, 73, 100, 101] could be more complex than CAMs due to several reasons [70]. In most countries conventional medicines are distributed with prescriptions that could be a hindering factor for the availability and accessibility of conventional medicines. In addition, high costs prevent the use of prescribed medicines, leading to many patients purchasing CAMs [65, 75]. Therefore, difficulties in availability-accessibility-affordability of conventional medicines could be driving forces that influence consumers to purchase CAMs.

## Discussion

This study introduces a novel model that used the best fit framework synthesis approach to comprehensively comprehend consumers’ purchase behavior of CAMs influenced by SMIs. Notably, to the best of the authors’ knowledge, this research represents the first empirical evidence illustrating the significant impact of SMI endorsements on consumers’ purchase intentions of CAMs. CAMPIM has several strengths. Firstly, the model provides a holistic overview and synthesis of the SMI endorsements (stimuli) that influence consumers’ psychological and cognitive processes (organism), leading to their intentions to purchase CAMs (response). Secondly, the development of the CAMPIM model involves a synergistic integration of two relevant theories, TPB and Source Credibility Theory, amalgamating existing research from diverse disciplines, thus yielding unique insights into the underlying mechanisms and deep perspectives governing consumers’ intentional behavior towards CAM purchases under the influence of SMIs. CAMPIM offers a more comprehensive understanding of the dynamic interplay between SMIs, consumers, and purchase intentions in the context of CAMs, paving the way for future research and evidence-based strategies in influencer marketing and CAM consumption.

Legislation around CAM use differs across different countries; where some countries regulate the manufacturing, distribution, and sale of CAMs like pharmaceutical products [109], but most governments do not regulate CAMs as medical or health products [110]. This lack of regulation allows manufacturers of CAMs to promote their products without a scientific check on the promotional content [111]. The endorsement of unregulated CAM products by SMIs on social media platforms raises concerns regarding potential harm to consumers. Using unregulated CAMs as adjunct or supplementary therapies could reduce efficacy, interactions, or side effects when combined with conventional medicines [112–114], posing risks to patients’ well-being. Despite the potential adverse effects of CAMs reported in numerous studies [115–119], the lack of statutory legislation for CAMs enables their endorsement on social media, similar to commercial products, without adhering to regulations related to health and well-being. Given the varied and ambiguous statutory legislation of CAMs worldwide, the use of SMIs to endorse these products on social media platforms has the potential to pose threats to public health. Policymakers urgently need to introduce legislation specifically addressing the marketing of CAMs on social media platforms. Evaluating the content of CAM endorsements should be the responsibility of governments and policymakers, ensuring the protection of consumers’ health and well-being.

Effectively utilizing the insights gathered from the CAMPIM model necessitates a comprehensive and targeted marketing approach to promote CAMs. As marketers in the healthcare industry embrace the use of SMIs to endorse CAMs on various social media platforms, it becomes paramount to establish clear and stringent guidelines governing their promotional activities [111]. A crucial finding of this study highlights the need for marketers to conduct rigorous evaluations of SMI-generated content and claims before endorsement. Marketers can ensure alignment with established regulatory standards and evidence-based practices by inspecting the accuracy, validity, and scientific basis of the information presented. This thorough vetting process is paramount to safeguarding public health by preventing the dissemination of misleading or false information that could potentially harm consumers. This combined approach ensures that the promotion of CAMs through SMIs is conducted responsibly and ethically, fostering consumer trust and confidence in these products.

In implementing a successful marketing approach for healthcare products, including CAMs, prioritizing transparency and disclosure are essential. Marketers must be explicit about any affiliations or relationships between SMIs and the CAM products they promote, as honesty and integrity are crucial to building and retaining consumer trust. In today’s discerning environment, authenticity and transparency are pivotal in fostering consumer confidence.

Considering the increasing trend of people using the internet for health-related information, including medicines, products, and CAMs [120–122], people tend to perceive unlicensed, non-professional SMIs as credible as licensed healthcare professionals when seeking health-related information [123]. Information provided by SMIs with no education about healthcare can jeopardize the health of those who follow them on social media. This opens up a novel role for healthcare professionals to leverage their expertise in the realm of social media. By providing knowledge, guidance, and counseling on CAMs and Over-the-Counter (OTC) medicines available for online purchase, healthcare professionals can optimize medicine usage and effectively manage diseases. Collaborating with reputable healthcare professionals and organizations further enhances the credibility of promotional efforts. Involving licensed practitioners and CAM experts in marketing campaigns strengthens the authenticity and trustworthiness of endorsements. Testimonials and endorsements from healthcare professionals, especially pharmacists, who vouch for the effectiveness and safety of CAMs wield significant persuasive power in building consumer confidence. This collaborative approach bridges the gap in credible health information on social media and empowers consumers to make informed decisions about their health and well-being. To capitalize on this opportunity, professional organizations should explore the development of new roles as healthcare SMIs. By leveraging their expertise, these professionals can deliver reliable health information about diseases, health-related products, medical devices, and CAMs. Implementing regulated digital services for providing health-related information ensures uniformity of content and the dissemination of accurate knowledge. This approach enhances consumer trust and empowers individuals to make informed decisions about their health and adhere to medical advice, contributing to improved public health outcomes.

Moreover, the involvement of healthcare professionals in mainstream media to deliver public health messages is not a new concept [124, 125]. However, in the era of digitalized health, new opportunities arise, such as designing services with healthcare professionals as SMIs. The COVID-19 pandemic has accelerated the development and implementation of digitalized healthcare worldwide, including remote digital health services, which have shown improvements in public health benefits [126–128]. With a growing interest in digitalized health services, understanding the factors influencing consumers’ perceptions of CAMs on digital platforms, as presented in CAMPIM, becomes crucial for implementing e-health services. Monitoring and managing medication therapy, counseling patients, and making informed decisions about treatment can be enhanced through these digital avenues, fostering better health outcomes for consumers.

### Further scope of the study

The medical research council (MRC) framework of complex interventions [129] could be used to explore contextual factors. Implementation science could be used to inform the development and implementation of patient counselling services by healthcare professionals in a digitalized format using social media platforms. Another advantage of this CAMPIM model is that it can give policy makers a baseline, for the required competencies of healthcare professionals, to deliver these kinds of services.

Considering its breadth, the CAMPIM may also be relevant and useful not just for enhancing new roles of healthcare professionals for policy makers to enable them to be more active on social media to protect public health, but also laying the foundations for marketers in the pharmaceutical sector to understand the complexity of SMIs’ influence on consumers’ intention to purchase CAMs. For example, if the aim is to decrease the risk of irrational use of CAMs, professional bodies could adopt new ways to reach consumers to influence their beliefs and attitudes on social media by increasing the argument quality of healthcare professional opinion leaders who are rather selected due to their similar background and values with consumers. Furthermore, pharmaceutical companies could tune to reach target audiences genuinely interested in exploring alternative healthcare options by effectively utilizing the insights captured from the CAMPIM.

### Limitations of the study

The CAMPIM has some limitations. One limitation is that the relationships among the constructs included in the model does not apply linearity in principle. Many constructs can affect and impact other constructs simultaneously and can positively or negatively impact the other constructs and may affect the behavior to purchase CAMs. It is also important to highlight that it is impossible to understand the correlations of these constructs within the model; for example, the reciprocal relationship between trustworthiness and expertise could not be shown in the model, if any. The authors thus propose future studies to evaluate the impact of constructs, mediators, and factors with each other and their overall impact on the purchase of CAM.

Another limitation was that as there was a lack of studies reporting the influence of SMIs on the purchasing behavior of CAM thus, the model relied on studies from different fields and was constructed by triangulating the findings. Although the model uses theory to understand how the constructs lead to the purchase intention, it differs widely from a realist evaluation and does not create any program theories. The model thus lacks the power to infer causality.

The model might need to undergo a rigorous overview by an expert panel of relevant stakeholders, including but not limited to consumers, current SMIs, and policy makers to validate and clarify concepts and to determine causal pathways among constructs, which authors are already planning to design a further study to evaluate the constructs of CAMPIM using stakeholders and expert panel with mixed methods. The model has the strength to be flexible in predicting the behavior of the purchase of CAM across different social media platforms. Still, the dynamics of each social media need to be accounted for in terms of producing an impact. Another feature of interest is that the sub-construct of ‘visuality’ in the model can be differently presented in various social media platforms. For example, the visuality of SMI would be presented differently than on Twitter, but the impact of visuality remains true in influencing the purchase behavior towards CAM. The dynamics of each platform may differ, and the model should be carefully examined and contextualized for developing new roles of health SMI’s across different platforms and implementing successful marketing approaches.

## Conclusions

This study presents a new and comprehensive model to understand factors influencing consumers’ purchase intention of CAMs endorsed by SMIs on social media platforms. The CAMPIM is a promising model that provides a holistic overview of social media factors influencing the purchase of CAMs. During the development of the CAMPIM model, it became evident that a versatile marketing approach that prioritizes transparency, targeted messaging, and collaboration with healthcare professionals is crucial for the responsible promotion of CAMs through SMIs. By adopting these evidence-based strategies, marketers can enhance consumer trust, empower informed decision-making, and promote the safe and effective use of CAMs in the digital era. However, the lack of regulation around CAMs highlights the need for urgent policy intervention to protect consumers from potential harm caused by unregulated endorsements. Implementing evidence-based strategies for enhancing new roles for healthcare professionals as SMIs will empower consumers to make informed decisions and promote the safe and responsible use of CAMs through social media platforms.

### Supplementary Information

**Additional file 1.**

**Additional file 2.**

## Data Availability

All relevant data are included in this manuscript. Furthermore, data set used in this study will be available upon request from the correspondence author.
