# Supplementary material for: A model of purchase intention of complementary and alternative medicines: the role of social media influencers’ endorsements
Source: BMC Complement Med Ther. 2023 Dec 5;23:439. doi: 10.1186/s12906-023-04285-1 (PMC10696731; doi:10.1186/s12906-023-04285-1)
Supplement: Supplementary file 1 — Additional file 1. [file 12906_2023_4285_MOESM1_ESM.docx]

Appendix 1. Keywords and MeSH terms used for searches

| MB | GG | AI |
| --- | --- | --- |
| - Complementary and alternative medicines - Complementary and alternative medicines systematic reviews - CAM systematic reviews - Complementary and alternative medicines scoping reviews - CAM scoping reviews | - Influencer marketing - Word-of-mouth - e-WOM (electronic word-of-mouth) - Social media influencer - Social media opinion leaders - Digital influencer - Online influencer - Online opinion-leader - Influencer endorsement - Celebrity endorsement - Social credibility - Influencer credibility - Social media influencer - Opinion leader credibility | - CAM - CAM complementary and alternative medicine - Complementary and alternative medicines - Complementary Therapies - Herbal medicines and products - Herbal medicines - Crude drugs - Herbals |
