# Supplementary material for: A model of purchase intention of complementary and alternative medicines: the role of social media influencers’ endorsements
Source: BMC Complement Med Ther. 2023 Dec 5;23:439. doi: 10.1186/s12906-023-04285-1 (PMC10696731; doi:10.1186/s12906-023-04285-1)
Supplement: Supplementary file 2 — Additional file 2. [file 12906_2023_4285_MOESM2_ESM.docx]

Supplementary table 2. The details of the theories and models identified

| Theory and Model | Included as a priori theory in the model | Details |
| --- | --- | --- |
| Dual Process Theory (DPT) | No | Describes analytic processing and heuristic processing that are encouraging people to focus on specific attributes and most dominant aspects of the target object being advertised respectively when making decisions. |
| Elaboration Likelihood Model (ELM) | No | Provides conceptualization of how consumers are persuaded to attitude change by promotional messages. |
| Information Acceptance Model (IAM) | No | Models the factors affect consumers to come to accept an information and plays a role in the purchase decision-making. |
| Self-Congruity Theory (SCT) | No | Illustrates the extent to which an individual perceives a product or brand as consistent with how they perceive themselves. |
| Social Influence Theory (SIT) | No | Describes the factors affecting individuals influenced by others. |
| Source Credibility Theory (SCT) | Yes | Explains how communication's persuasiveness is affected by the perceived credibility of the source of the communication. |
| Stimulus-Organism-Response Model (SOR) | No | Describes the connection between stimuli (such as external factors) that will affect organisms (cognition and emotion of people) and the response people have to the stimulus (such as behavior). |
| Technology Acceptance Model (TAM) | No | Describes the factors affecting the adoption of new technology based on customer attitudes. |
| Theory of Planned Behaviour (TPB) | Yes | Assumes that behaviors are influenced by intentions, which are determined by three factors: attitudes, subjective norms, and perceived behavioral control. |
| Theory of Reasoned Action (TRA) | No | Explains the relationship between attitudes and behaviors within human action. |
| Unified Theory of Acceptance And Use of Technology (UTUAT) | No | Aims to explain user intentions to use an information system and subsequent usage behavior. |
| Uses and Gratifications Theory (UGT) | No | Adapts a functionalistic approach to communications and media, and states that media's most important role is to fulfill the needs and motivations of the audience. |
